# Neutrophil extracellular traps promote growth of lung adenocarcinoma by mediating the stability of m6A‐mediated SLC2A3 mRNA‐induced ferroptosis resistance and CD8(+) T cell inhibition

**DOI:** 10.1002/ctm2.70192

**Published:** 2025-01-26

**Authors:** Li Xu, Yi Kong, Kang Li, Jia Li, Fang Xu, Yan Xu, Shuzhi Liang, Bolin Chen

**Affiliations:** ^1^ The Second Department of Thoracic Oncology The Affiliated Cancer Hospital of Xiangya School of Medicine, Central South University/Hunan Cancer Hospital Changsha Hunan Province P.R. China

**Keywords:** lung adenocarcinoma, m6A, neutrophil extracellular traps, SLC2A3, tumour immune microenvironment

## Abstract

To investigate the potential mechanisms underlying neutrophil extracellular traps (NETs) confer ferroptosis resistance and CD8(+) T cell inhibition in lung adenocarcinoma (LUAD). By the intravenous injection of LLC cells into the tail vein, a LUAD mouse model was created. Phorbol‐12‐myristate‐13‐acetate (PMA) stimulated neutrophils to facilitate NETs formation and combined with NETs inhibitor DNase I to explore NETs mechanism on LLC cell proliferation, migration, ferroptosis resistance, and CD8(+) T cell activity. CitH3, myeloperoxidase (MPO), cell‐free DNA, and MPO‐DNA levels in LUAD were increased, indicating an increase in NETs formation in LUAD. PMA promoted NETs formation in tumours of mice, increased the number of CD3(+)CD4(+) T cells, decreased perforin, granzyme A, granzyme B, IFNγ, and TNF‐α levels, and promoted LUAD growth and the number of lung tumour nodules, indicating that PMA promoted NETs formation, reduced the activity of CD8(+)T cells, and promoted LUAD growth. DNase I partially reversed the effects of PMA. NETs promoted LLC cell proliferation and migration, while DNase I reversed NETs effects. Erastin inhibited LLC cell proliferation and migration and promoted ferroptosis. NETs partially reversed Erastin effects. Further results showed that NETs promoted LLC cell proliferation and migration and inhibited ferroptosis by promoting YTHDF2‐mediated SLC2A3 mRNA degradation. Sh‐YTHDF2 partially reversed the effect of NETs on LLC cells, whereas si‐SLC2A3 partially reversed sh‐YTHDF2 effects on LLC cells. In addition, NETs inhibited LLC cell ferroptosis by inhibiting CD8(+) T cell activity. Sh‐YTHDF2 and DNase I inhibited NETs formation in tumours, increased the activity of CD8(+) T cells and inhibited LUAD growth. Our results suggested that NETs promoted the growth of LUAD through inhibiting ferroptosis and CD8(+) T cell activity by promoting YTHDF2‐mediated SLC2A3 mRNA degradation.

## Graphical abstract

NETs promote metastatic growth of LUAD by promoting YTHDF2‐mediated SLC2A3 mRNA degradation, inhibiting ferroptosis and CD8(+) T cell activity.

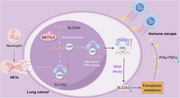

## 1 INTRODUCTION

Lung cancer has emerged as a formidable public health concern, accounting for the highest number of mortalities globally.^1^ Lung adenocarcinoma (LUAD) represents the histological type of lung cancer most frequently encountered in clinical practice.^2^ Notwithstanding advancements in radiotherapy, chemotherapy, and immunotherapy for lung cancer, the 5‐year survival rate remains suboptimal.^3^ Further investigation of the mechanisms underlying the development of LUAD may help identify novel therapeutic targets.

Neutrophil extrinsic traps (NETs) were originally thought to be a network of DNA histones and proteins released by activated neutrophils that dominate the innate immune response mediated by neutrophils.^4^ Increased NETs promote the metastatic potential of hepatocellular carcinoma by triggering tumour inflammatory responses.^5^ Cathepsin C facilitates the metastasis of breast and lung cancers by regulating neutrophil infiltration and NETs formation.^6^ NETs promote non‐small cell lung cancer (NSCLC) metastasis by activating nuclear factor‐kappaB by inhibiting MIR503HG inflammasome pathway.^7^ The tumour microenvironment (TME) is an ecosystem composed including tumour cells, immune cells, and cancer‐associated fibroblasts.^8^ NETs promote tumour growth by inhibiting T cell responses.^9^ NETs can regulate the immune response by activating COX‐2 through Toll‐like receptor 2, thereby enhancing the metastatic ability of gastric cancer cells.^10^ Prior research has demonstrated that NETs are a significant contributor to the TME. The precise molecular mechanisms by which NETs operate within the TME remain poorly understood.

Ferroptosis represents a regulated form of cell death whereby the excessive accumulation of iron‐dependent lipid peroxides, which has recently been linked to LUAD.^11^ The ferroptosis‐related gene SLC2A3 is associated with tumour immune response and patient prognosis.^12^, ^13^ The SLC2A3 gene encodes for the neuronal glucose transporter 3 (GLUT3), a transmembrane protein responsible for facilitating the diffusion of glucose across plasma membranes in response to intracellular and extracellular cues.^14^ SLC3A2, as a heavy chain subunit, and light chain subunit SLC7A11 form a cystine/glutamate transporter (System Xc‐), which is principally accountable for the cellular uptake of cystine in exchange for intracellular glutamate.^15^ Nevertheless, the precise mechanism by which SLC2A3 exerts its effects in LUAD remains unclear. N6‐methyladenosine (m6A) is a universal modification of RNA molecules among many epigenetic changes and has become an important regulator of cancer progressions.^16^ METTL3 plays a dual role in LUAD, acting as a tumour promoter and ferroptosis inhibitor through stabilisation of SLC7A11 m6A modification.^17^ Furthermore, m6A modification has been implicated in numerous facets of LUAD and facilitates the formation of TME.^18^ Bioinformatics analysis showed that m6A and NETs‐related lncRNA models could predict liver cancer prognosis and immune landscape.^19^ NETs serve as mediators of m6A modification, thereby modulating sepsis‐associated acute lung injury through the activation of ferroptosis in alveolar epithelial cells. However, the biological significance and underlying mechanism of NETs‐mediated ferroptosis through m6A in LUAD remain elusive.

This study aimed to investigate the potential molecular mechanism by which CD8(+) T cell inhibition in LUAD might be facilitated by NETs‐mediated ferroptosis via the m6A pathway.

## 2 MATERIALS AND METHODS

### 2.1 Clinical samples

LUAD (*n* = 10), paracancer tissues (*n* = 10), and serum samples from LUAD (*n* = 10) and normal (*n* = 10) patients were obtained from the Hunan Cancer Hospital. A comprehensive overview of the clinical characteristics of the patients is provided in Supplementary Table 1. This study was approved by the Medical Ethics Committee of Hunan Cancer Hospital (Number: SBQLL‐2022‐113). All data and materials were performed following the Declaration of Helsinki and conformed to relevant aspects of the ARRIVE guidelines. All the information about the study will be fully explained to the subjects by the researchers. All patients signed written informed consent. Informed consent was obtained from the participants.

### 2.2 Animal model

The mouse model was created through the following methodology: C57BL6/J mice were sacrificed three weeks after injection with labelled/unlabelled‐a green fluorescent protein (GFP) Lewis lung cancer cells (LLC, 3×10^5^ cells) through the tail vein.^20^ To ascertain Phorbol‐12‐myristate‐13‐acetate (PMA)‐induced NETs impacts on lung cancer, PMA (50 ng/g mouse body weight) was intraperitoneally administered 1 day before cancer cell injection. The mice were administered an intraperitoneal injection of DNase I (100 U/mouse) on a daily basis.^21^ All experimental procedures and animal handling were performed with the approval of the Animal Care and Use Committee of the Hunan Cancer Hospital (Number: 2022–084), in accordance with the National Institutes of Health Guide for the Care and Use of Laboratory Animals.

### 2.3 Cell culture and transfection

LLC cells (AW‐CCM076, Abiowell, China) were cultured in F‐12K medium and DMEM. According to the manufacturer's instructions, plasmid transfection was performed in LLC cells using Lipofectamine 2000 (Invitrogen).^22^ YTHDF2‐WT and YTHDF2‐MUT were used for the transfection. The shRNA sequence targeting YTHDF2 (sh‐YTHDF2) was as follows: sh‐YTHDF2#1, 5′‐TACTGATTAAGTCAGGATTAA‐3′; sh‐YTHDF2#2, 5′‐ATGGATTAAACGATGATGAT‐3′. The stimulation of neutrophils with PMA (100 nM) was conducted for 4 h to ascertain the induction of NETs. Neutrophils were treated with DNase I to observe their effects on NETs. NETs were digested with 1.5 U DNase I.^23^ Erastin is a classical ferroptosis inducer.^24^ To further explore NETs‐mediated ferroptosis effects on LLC cells, NETs and LLC cells (2:1) were cocultured for 12 h.^7^, ^25^ LLC cells underwent simultaneous treatment with erastin (10 µM/mL) and NETs for 12 h.^26^, ^27^

### 2.4 NETs formation and visualisation

Isolated neutrophils were suspended in RPMI 1640 and then inoculated in a 6‐well plate. The stimulation of neutrophils with PMA (100 nM) was conducted for 4 h at 37°C and 5% CO_2_ to ascertain the induction of NETs. NETs were then collected according to a multi‐step centrifugation protocol recommended in a previous study.^28^ To determine the number of neutrophils, an equivalent number (1 × 10⁷) was cultured in a 6‐well plate with the addition of PMA (100 nM) for 4 h, allowing for the generation of NETs. Subsequently, the supernatant was extracted and washed twice gradually and gently, ensuring that the NETs were not compromised to remove any impurities. The supernatant was collected and subjected to centrifugation to purify the NETs, which were stored at −80°C for subsequent experiments.

### 2.5 Bioinformatics

The initial NSCLC dataset was procured from the TCGA database (https://gdc.nci.nih.gov/). The ensuing data analysis was conducted with the assistance of the R × 64.3.5.3 software program. The differentially expressed genes in normal tissue and tumour tissue were analysed using the edgeR package in R language, and the screening parameters were set as |logFC| > .4 and adjusted *p*‐value < .05. The genes associated with ferroptosis were obtained from the database (FerrDhttps://doi.org/10.1093/nar/gkac935). The intersection gene between the m6A‐modified gene and the YTHDF2 targeted gene was obtained, and the intersection gene with the ferroptosis‐related gene was obtained.

### 2.6 RNA binding protein immunoprecipitation assays (RIP)

The Magna RIP kit, produced by Merck Millipore, was employed. The exponentially growing cells were subjected to transfection using a 10 µg YTHDF2 plasmid, placed on a 10 cm plate for 48 h, and cleaved with RIP lysis buffer. The RIP lysate was then centrifuged again. Magnetic beads were prepared using an anti‐YTHDF2 antibody or anti‐IgG antibody coupling. The remaining supernatant and antibody bead‐antibody complex were added to RIP immunoprecipitation buffer and incubated at 4°C overnight. A solution containing Protease K and 10% SDS was prepared and incubated at 55°C for 30 min to digest protein. The RNA was purified by precipitation. qRT‐PCR was used to assess specific gene expression.

### 2.7 Methylated RNA immunoprecipitation sequencing (MeRIP‐seq)

m6A modifications in specific mRNA transcripts were detected using the piQuik™CUT&RUN m6A RNA enrichment kit. First, 50 µg of total RNA was isolated using TRIzol reagent. Next, the RNA sequence fragment containing m6A target was cut, bound to the magnetic bead containing the m6A antibody, and pulled down. The enriched RNA was released, purified, and eluted. We designed YTHDF2 m6A binding protein‐specific primers according to SRAMP information (http://www.cuilab.cn/sramp). Finally, changes in m6A modification of some genes were detected using qRT‐PCR. The primers utilised are enumerated in Table 2.

### 2.8 RNA stability determination

The cells were subjected to transfection with either sh‐NC, sh‐YTHDF2‐1 or sh‐YTHDF2‐2 plasmid for 48 h. Subsequently, treatment was initiated with 5 µg/mL actinomycin D (ThermoFisher, USA) for 0, 1, 2, and 4 h. Trizol extracted the total RNA from these cells. The relative expression of specific genes at a given time point was analysed using qRT‐PCR. The degradation rate of mRNA was calculated as the ratio of mRNA levels at the specified time to mRNA levels at 0 h. The degradation curve of specific genes were plotted according to the degradation rate.

### 2.9 RNA pull‐down

The RNA antisense purification kit used in this experiment was procured from BersinBio (Guangzhou, China). Briefly, 2 × 10^7^ cells were lysed in lysate buffer containing protease and RNase inhibitors. The resulting solution was subjected to centrifugation at 16 000 *g*. Subsequently, 40 pmol biotinylated SLC2A3 probe was incubated with the supernatant at 37°C for 3 h. Streptavidin beads (20 µL) were subjected to a washing procedure, following which they were hybridised with the supernatant for 30 min. The protein concentrate was bound to beads for elution prior to the western blot analysis.

### 2.10 Statistics and analysis

The data are represented as means ± standard deviation (SD). The data were processed using GraphPad software (GraphPad) and analysed using Student's *t*‐test or one‐way analysis of variance (ANOVA). In all cases, the level of significance was set at a value of *p* ≤.05.

## 3 RESULTS

### 3.1 NETs formation was elevated in LUAD patients

**FIGURE 1:**
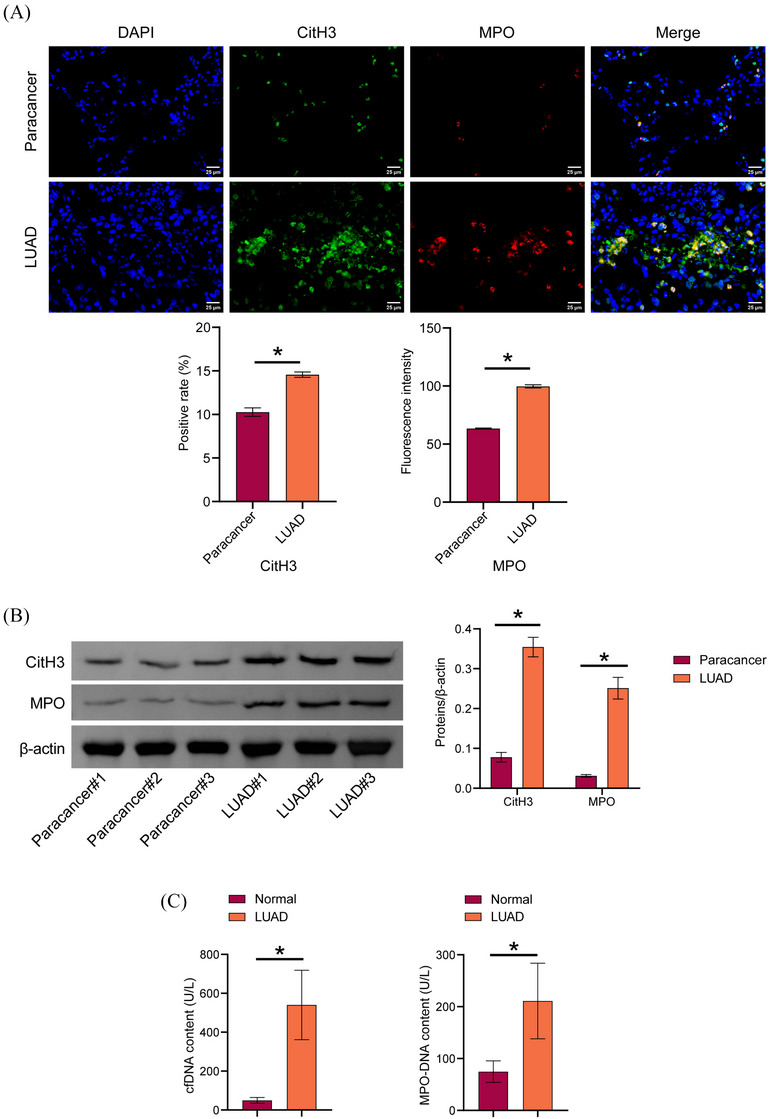
Formation of NETs in LUAD and extraction of primary neutrophils. (A) IF detection of CitH3 and MPO localization in lung cancer tissue. (B) CitH3 and MPO proteins expression in LUAD tissues. (C) The biochemical kit detected the cfDNA and MPO‐DNA content in the serum of LUAD patients. *p < .05.

To explore NETs levels in LUAD, CitH3 and MPO expression in LUAD tissues was detected by IF and Western blot. CitH3 and MPO expression were increased in LUAD tissues compared to paracancer tissues. We observed web‐like structures characteristic of NETs in LUAD tissues (Figure 1A and B). Serum cfDNA and MPO‐DNA levels were increased in LUAD patients compared to those in the normal group (Figure 1C). These findings indicated an elevation in NET levels in patients with LUAD.

### 3.2 NETs promoted LUAD growth and inhibited CD8(+) T cell activity

**FIGURE 2:**
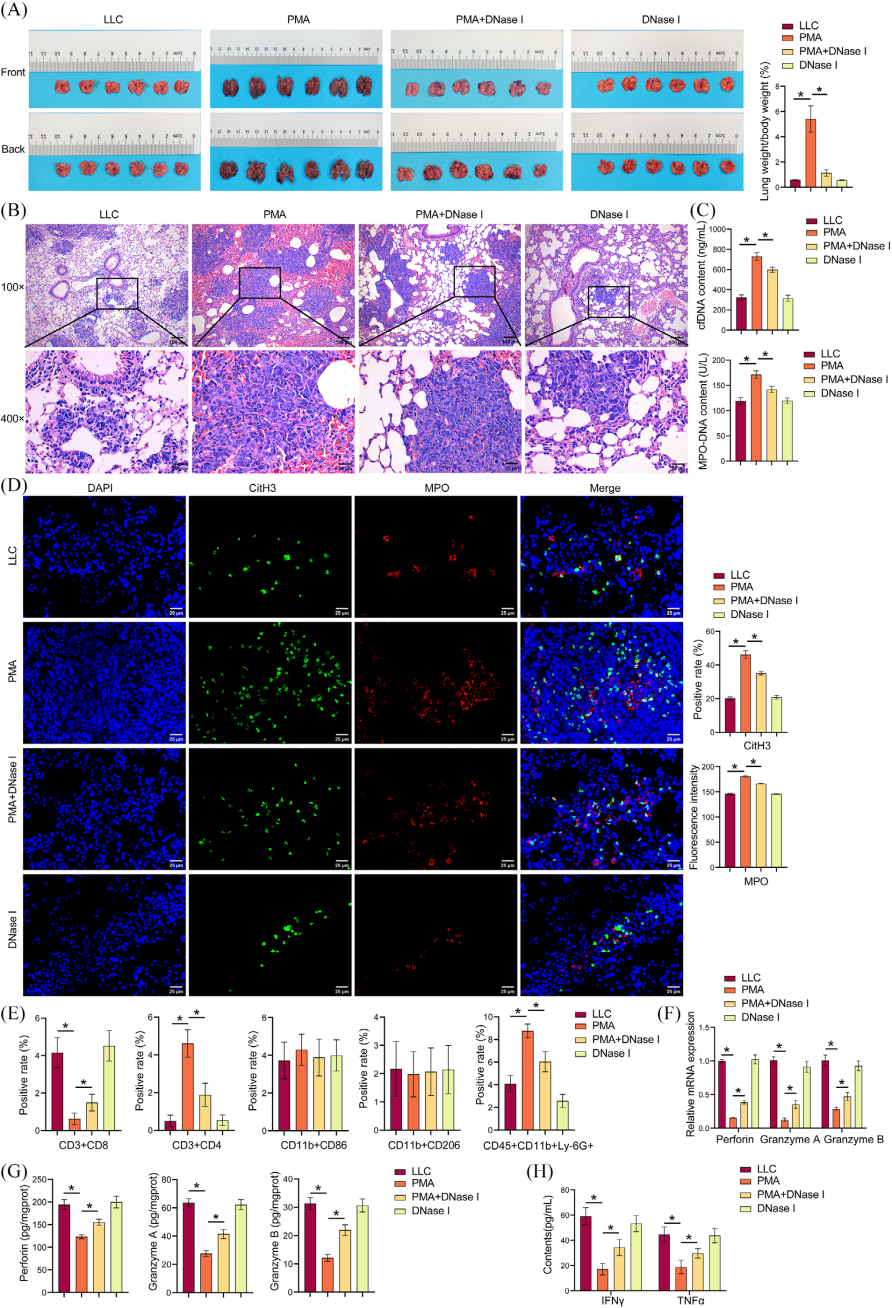
NETs promoted LUAD growth and inhibited CD8(+) T cell activity. (A) Lung tumour images of mice and lung weight/body weight. (B) H&E staining analysis of tissue morphology. (C) Biochemical kit analysis of cfDNA and MPO‐DNA content. (D) IF analysis of CitH3 and MPO expression in lung cancer tissues. (E) A flow cytometry analysis was conducted on CD3(+)CD8(+)T cells, CD3(+)CD4(+)T cells, CD45(+)CD11b(+)Ly‐6G(+)neutrophils, M1 macrophages (CD11b(+)CD86(+)), and M2 macrophages (CD11b(+)CD206(+)). (F) qRT‐PCR assay and (G) ELISA detection of perforin, granzyme A and granzyme B expression. (H) ELISA detection of IFNγ and TNF‐α. *p < .05.

Prior research has demonstrated that NETs are intimately linked with tumour progression and metastasis, including breast cancer lung metastasis,^29^ liver cancer lung metastasis,^30^ and NSCLC metastasis.^7^ The underlying molecular mechanism of NETs in LUAD remains to be elucidated. Next, NETs effects on LUAD growth and CD8(+) T cell activity were investigated. PMA increased the positive rate of GFP in the lung tissue, and DNase I reversed the stimulatory effect of PMA on the positive rate of GFP (Figure S2B). PMA increased the lung weight‐to‐body weight ratio in mice. DNase I partially reversed PMA effects and reduced lung weight‐to‐body weight ratio (Figures 2A). PMA increased the number and size of pulmonary nodules, while DNase I reversed this effect (Figure 2B). PMA increased cfDNA and MPO‐DNA contents, while DNase I prevented this effect and decreased cfDNA and MPO‐DNA contents (Figure 2C). PMA increased CitH3 and MPO expression, and DNase I treatment reversed the stimulatory effect of PMA on CitH3 and MPO expression (Figure 2D). The results demonstrated that PMA induced an elevation in NETs in vivo, while DNase I partially reversed the increase. PMA reduced the number of CD3(+)CD8(+)T cells and increased the number of CD3(+)CD4(+)T cells and CD45(+)CD11b(+)Ly‐6G(+)neutrophils. This effect was prevented by DNase I. No notable discrepancy was observed in the number of M1 and M2 macrophages (Figures 2E and S1A). After sorting tumour CD8(+)T cells using magnetic bead, perforin, granzyme A, and granzyme B expression were assessed by qRT‐PCR and ELISA. PMA inhibited perforin, granzyme A, and granzyme B expression, which reversed by DNase I (Figure 2F and G). PMA downregulated IFNγ and TNF‐α levels in the serum. Compared to the PMA group, IFNγ and TNF‐α levels in the PMA+DNase I group were upregulated (Figure 2H). Our results suggested that NETs promoted LUAD growth and inhibited CD8(+) T cell activity.

### 3.3 NETs promoted proliferation, migration, and ferroptosis resistance of LLC cells

**FIGURE 3:**
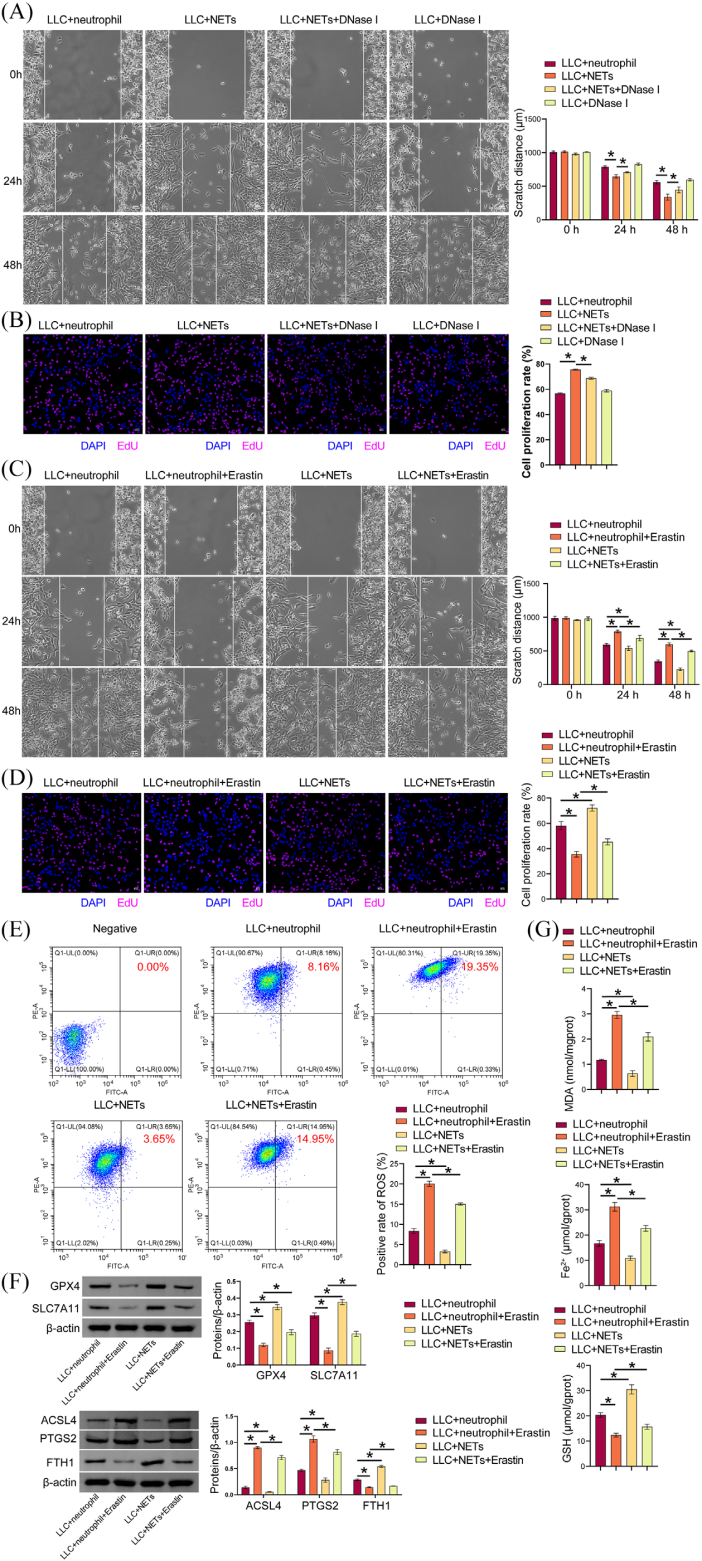
**NETs effects on proliferation, migration, and ferroptosis resistance of LLC cells**. (A) Cell migration capacity. (B) EdU was used to evaluate cell proliferation. (C) Cell migration capacity. (D) EdU was employed for the purpose of detecting cell proliferation. (E) Lipid ROS was determined through the use of C11 BODIPY 581/591. (F) GPx4, SLC7A11, FTH1, ACSL4, and PTGS2 proteins expression. (G) Biochemical kits were used to detect MDA, total Fe^2+^, and GSH levels. *p < .05.

Compared to the LLC+neutrophil group, the LLC+NETs group demonstrated enhanced cell proliferation and migration, which were decreased in the LLC+NETs+DNase I group compared to the LLC+NETs group (Figure 3A and B). These findings demonstrated that NETs facilitated cell proliferation and migration. Prior investigations have indicated a correlation between NETs and resistance to ferroptosis during tumour progression.^31^, ^32^ Therefore, we explored the effect of NETs on ferroptosis resistance in LUAD. Compared with the LLC+neutrophil group, the LLC +neutrophil+Erastin group had lower cell proliferation and migration abilities, which were enhanced in the LLC+NETs group. NETs reversed erastin effects on LLC cell proliferation and migration (Figure 3C and D). Erastin increased cellular lipid ROS in LLC cells. Compared with the LLC+neutrophil group, cell lipid ROS in the LLC+NETs group was decreased. Cellular lipid ROS decreased in the LLC+NETs+Erastin group compared with the LLC +neutrophil+Erastin group (Figure 3E). A comparison of the LLC+neutrophil group with the LLC+neutrophil+Erastin group revealed a decrease in GPX4, SLC7A11, and FTH1 expression in the latter, while showing an increase in ACSL4 and PTGS2 levels. A comparison of the LLC+neutrophil group with the LLC+NETs group revealed an increase in GPX4, SLC7A11, and FTH1 levels in the latter, while showing a decrease in ACSL4 and PTGS2 levels. A comparison of the LLC+neutrophil+Erastin group with the LLC+NETs+Erastin group revealed an increase in GPX4, SLC7A11, and FTH1 levels in the latter, while showing a decrease in ACSL4 and PTGS2 levels (Figure 3F). Erastin elevated MDA and total Fe^2+^ levels in LLC cells, accompanied by a reduction in GSH levels. In comparison to the LLC+neutrophil group, MDA and total Fe^2+^ levels were diminished in the LLC+NETs group, while the GSH content was elevated. MDA and total Fe^2+^ levels were decreased, while those of GSH were increased in the LLC+NETs+Erastin group in comparison to the LLC+neutrophil+Erastin group (Figure 3G). Our results suggested that NETs promoted LLC cell proliferation, migration, and ferroptosis resistance.

### 3.4 NETs promoted LLC cell proliferation, migration, and ferroptosis resistance through YTHDF2

**FIGURE 4:**
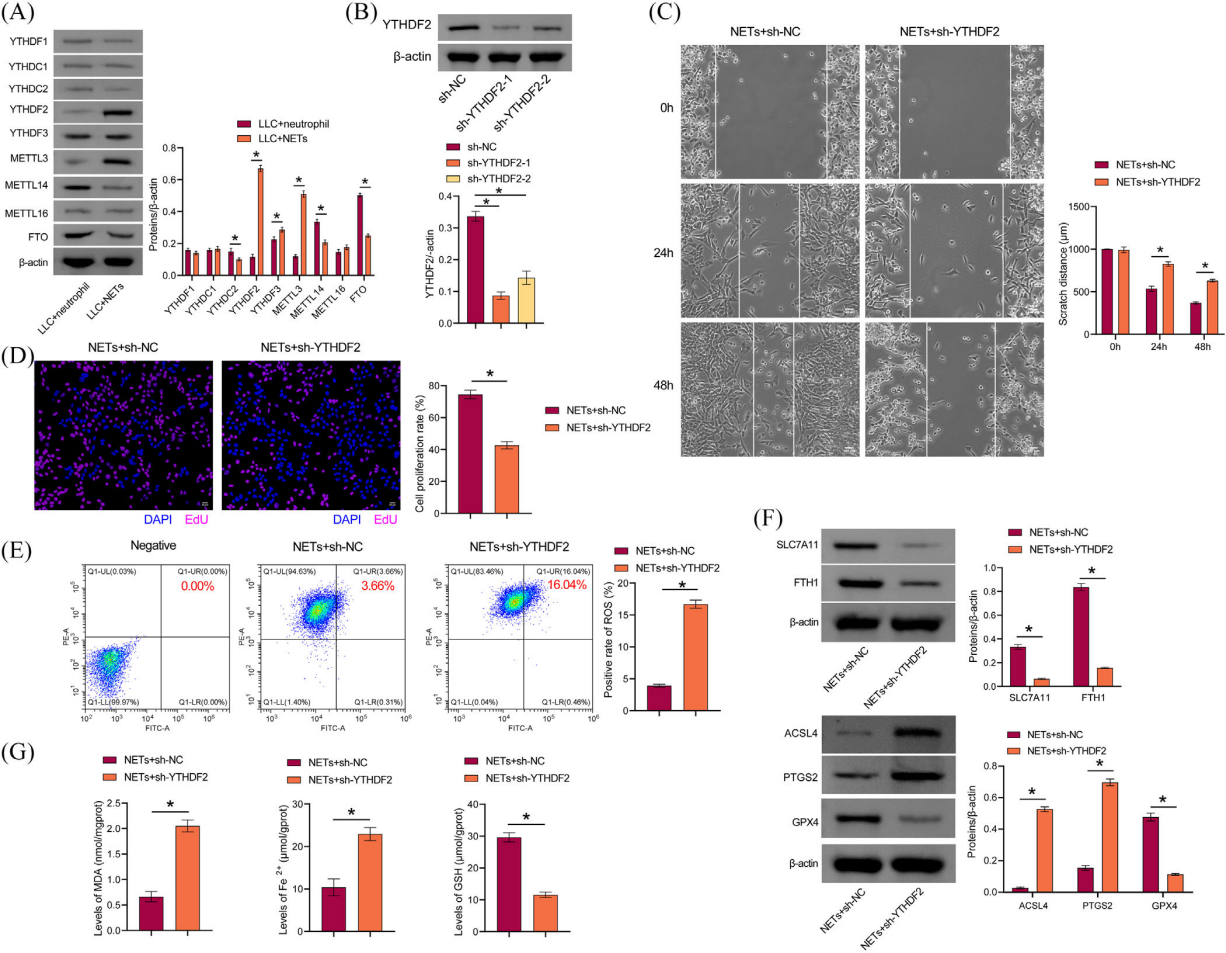
**NETs enhanced LLC cell proliferation, migration, and ferroptosis resistance through YTHDF2**. (A) Western blot analysis of YTHDF1, YTHDF2, YTHDF3, YTHDC1, YTHDC2, methyltransferase METTL3, METTL14, METTL16, and FTO expression. (B) YTHDF2 protein expression. (C) Scratch detection of cell migration capacity. (D) EdU was employed for the purpose of detecting cell proliferation. (E) The presence of lipid ROS was determined through the use of C11 BODIPY 581/591. (F) GPx4, SLC7A11, FTH1, ACSL4, and PTGS2 proteins expression. (G) Biochemical kits were used to detect MDA, total Fe^2+^, and GSH levels. *p < .05.

m6A methylation is the most prevalent and conserved mRNA modification in eukaryotes.^33^ Neutrophils in breast cancer can mediate m6A methylation of WTAP‐dependent ENO1 to promote glycolysis in breast cancer.^34^ NETs play a role in mediating m6A modification, which in turn regulates sepsis‐associated acute lung injury through the activation of ferroptosis.^35^ Therefore, we explored whether NETs modulated LLC cell proliferation, migration, and ferroptosis resistance through m6A methylation. We found that compared with the LLC+neutrophil group, YTHDF2, YTHDF3, and METTL3 levels in LLC cells of the LLC+NETs group were increased, while YTHDC2, METTL14, and FTO levels were decreased. YTHDF2 showed the most significant difference (Figure 4A), and YTHDF2 was selected for further study. A comparison of the sh‐NC group with the sh‐YTHDF2‐1 and sh‐YTHDF2‐2 groups revealed a decrease in YTHDF2 levels, indicating that the cells were successfully transfected with sh‐YTHDF2 (Figure 4B). We selected sh‐YTHDF2‐1, which had a higher transfection efficiency, for the follow‐up study. sh‐YTHDF2 inhibited the proliferative and migratory capacity of LLC cells treated with NETs (Figure 4C and D). In addition, sh‐YTHDF2 reduced the migratory capacity of LLC cells (Figure S1B). Sh‐YTHDF2 promoted lipid ROS production in LLC cells treated with NETs (Figure 4E). Sh‐YTHDF2 inhibited GPX4, SLC7A11, and FTH1 expression in LLC cells treated with NETs, while promoting ACSL4 and PTGS2 levels (Figure 4F). Sh‐YTHDF2 promoted MDA and total Fe^2+^ in LLC cells treated with NETs while decreasing GSH levels (Figure 4G). These findings indicated that NETs facilitated LLC cell proliferation, migration, and ferroptosis resistance via YTHDF2.

### 3.5 Screening and validation of downstream targets for the effect of NETs on YTHDF2 expression

**FIGURE 5:**
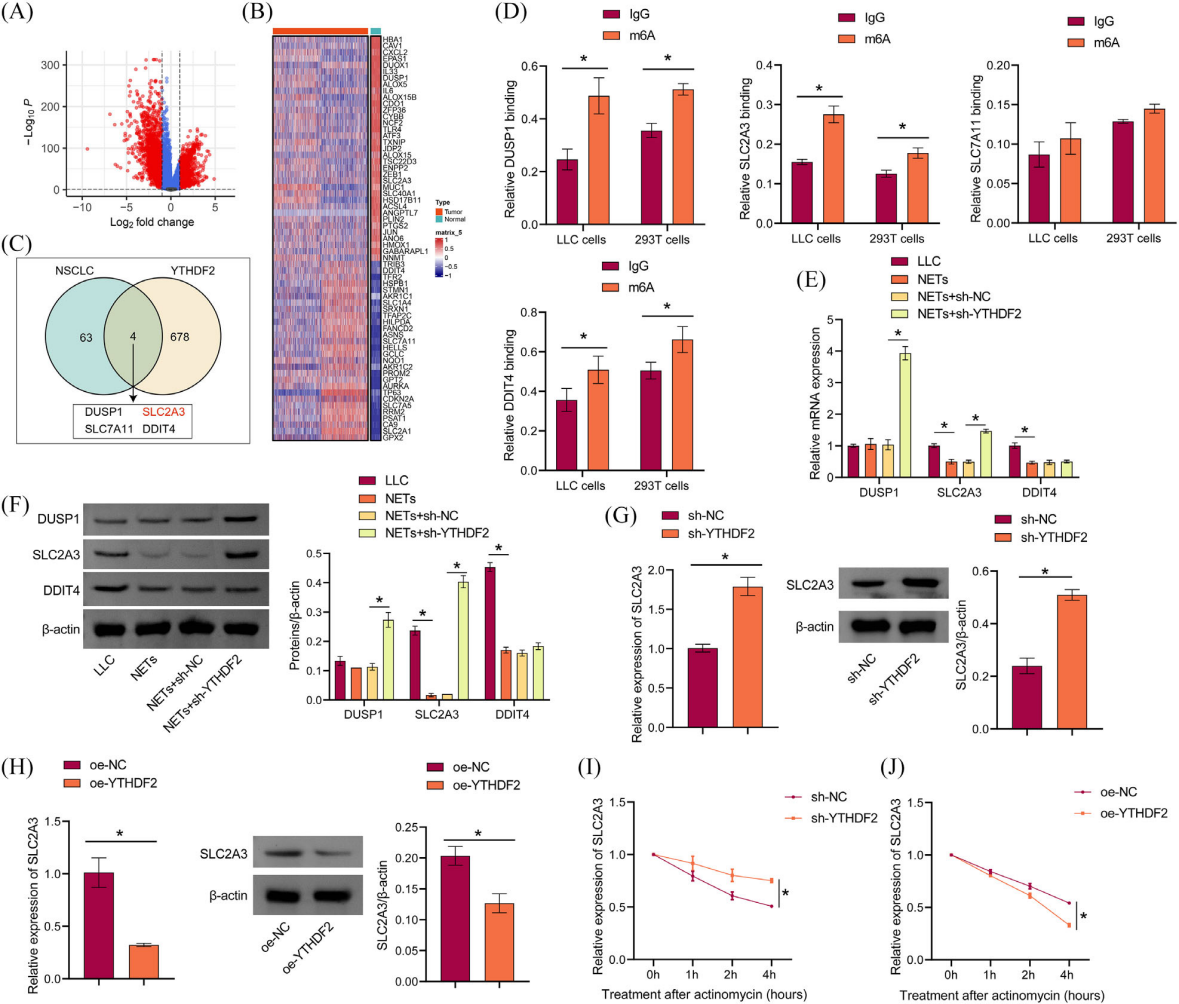
Screening and verification of downstream targets of NETs effect on YTHDF2 expression. (A) Volcano maps showed differentially expressed genes in NSCLC. (B) Heat maps revealed genes related to ferroptosis. (C) Venn diagram shows the intersection factors of 678 m6A modified genes with YTHDF2‐binding proteins and 63 ferroptosis‐related genes. (D) The relative enrichment folds of DUSP1, SLC2A3, SLC7A11, and DDIT4 mRNA correlated with YTHDF2. (E and F) DUSP1, SLC2A3, and DDIT4 mRNA and protein expression. (G and H) SLC2A3 mRNA and protein expression. (I and J) SLC2A3 mRNA stability analysis in LLC cells after knockdown or overexpression YTHDF2 with actinomycin D (5 µg/mL) for 0 h, 1 h, 2 h, and 4 h. *p < .05.

Bioinformatics analysis was performed to screen potential targets for NETs effects on the regulation of YTHDF2 expression. The volcano map shows the differentially expressed genes in NSCLC (Figure 5A). The heat map shows genes associated with ferroptosis (Figure 5B). According to previous studies,^36^ 678 genes that overlapped with m6A markers and bound to YTHDF2 were obtained. A total of 63 genes were identified by the intersection of differentially expressed NSCLC genes and ferroptosis‐related genes. Two genes were intersected, and four genes were obtained: DUSP1, SLC2A3, SLC7A11, and DDIT4 (Figure 5C). The RIP results showed that DUSP1, SLC2A3, and DDIT4 were combined with YTHDF2 (Figure 5D). Western blot results showed that NETs inhibited SLC2A3 expression. Sh‐YTHDF2 interrupted this effect and promoted SLC2A3 expression (Figure 5E and F). Moreover, sh‐YTHDF2 promoted SLC2A3 expression, and overexpression of YTHDF2 inhibited SLC2A3 expression (Figure 5G and H). Next, we used SRAMP (http://www.cuilab.cn/sramp) to predict that YTHDF2 might modify SLC2A3 mRNA m6A loci (Figure S3H). In the presence of actinomycin D, a drug that blocks RNA synthesis, we found that YTHDF2 knockdown inhibited SLC2A3 mRNA degradation, while YTHDF2 overexpression promoted SLC2A3 mRNA degradation (Figure 5I and J). In conclusion, we found that YTHDF2 promoted SLC2A3 mRNA degradation through m6A modification.

It has been reported that YTHDF2 relies on hydrophobic residues W432 and W486 as m6A ‘readers’ in its carboxy‐terminal YTH domain.^37^ Catalytic inactivation mutants of YTHDF2 (W432A and W486A) were constructed (Figure S3A). Western blot results showed that exogenous expression of WT‐YTHDF2, but not catalytically inactivated mutants, alleviated the YTHDF2‐induced decline in SLC2A3 mRNA stability and protein levels, while catalytically inactivated mutants had no such effects (Figure S3B and C). RNA pull‐down verified the binding of SLC2A3 mRNA to YTHDF2, the expression of YTHDF2 was reduced after mutation (Figure S3D and E). Si‐METTL3 decreased METTL3 expression while increasing SLC2A3 expression in LLC cells (Figure S3F). The interplay between METTL13 and SLC2A3 was diminished by si‐METTL3 (Figure S3G). Our data suggested that YTHDF2 reduced SLC2A3 mRNA stability in LLC cells.

### 3.6 NETs promoted ferroptosis resistance of LLC cells through the YTHDF2‐SLC2A3 axis

**FIGURE 6:**
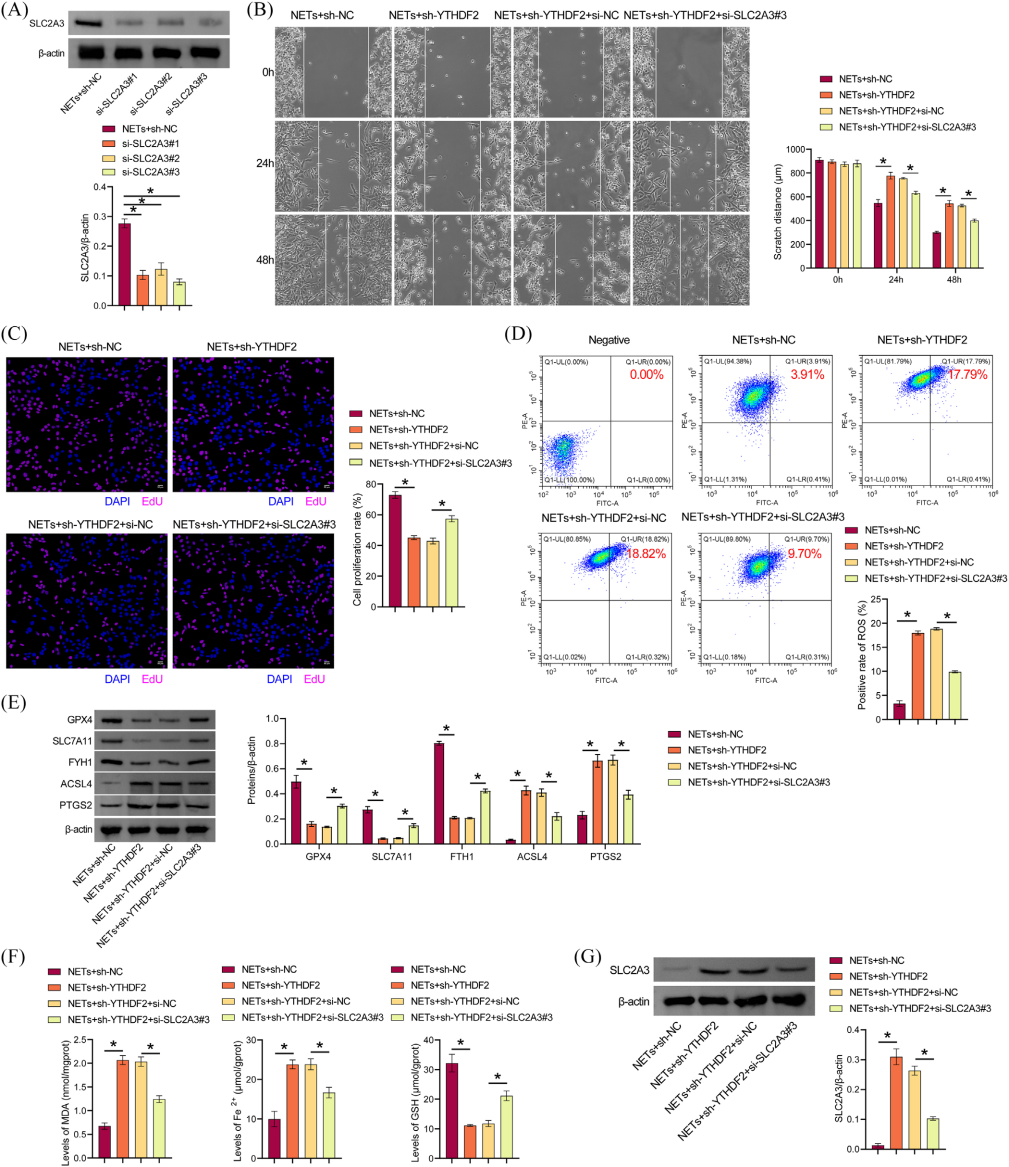
NETs promoted ferroptosis resistance in LLC cells via the YTHDF2‐SLC2A3 axis. (A) Knockdown SLC2A3 transfection efficiency verification. (B) Scratch detection of cell migration capacity. (C) EdU was applied for examining cell proliferation. (D) The presence of lipid ROS was determined through the use of C11 BODIPY 581/591. (E) GPx4, SLC7A11, FTH1, ACSL4, and PTGS2 proteins expression. (F) Biochemical kits were used to assess MDA, total Fe^2+^, and GSH levels. (G) Western blot analysis of SLC2A3 expression. *p < .05.

Next, the mechanism underlying NETs' function in LLC cells was explored in vitro. Western blot results showed that si‐SLC2A3 transfection was successful, and si‐SLC2A3 inhibited SLC2A3 expression in LLC cells (Figure 6A). We selected si‐SLC2A3#3 with the highest transfection efficiency for the follow‐up study. Sh‐YTHDF2 inhibited the proliferative and migratory capacity of LLC cells treated with NETs, which were reversed by the si‐SLC2A3 group (Figure 6B and C). Sh‐YTHDF2 increased lipid ROS in LLC cells, which were reversed by si‐SLC2A3 (Figure 6D). Compared with the NETs+sh‐NC group, GPX4, SLC7A11, and FTH1 levels in LLC cells of the NETs+sh‐YTHDF2 group were inhibited, while ACSL4 and PTGS2 levels were increased. Si‐SLC2A3 partially revoked the inhibitory effect of NETs+sh‐YTHDF2 on GPX4, SLC7A11, and FTH1 expression in LLC cells and the stimulatory effect of NETs+sh‐YTHDF2 on ACSL4 and PTGS2 levels (Figure 6E). Compared with the NETs+sh‐NC group, MDA and total Fe^2+^ levels in LLC cells of the NETs+sh‐YTHDF2 group were upregulated, while GSH levels were downregulated. Si‐SLC2A3 partially interrupted the promotion of MDA and total Fe^2+^ levels and the inhibition of GSH in NETs+sh‐YTHDF2LLC cells (Figure 6F). Compared with NETs+sh‐NC group, SLC2A3 expression was increased in LLC cells of NETs+sh‐YTHDF2 group, and si‐SLC2A3 partially revoked the promoting effect of SLC2A3 expression in NETs+sh‐YTHDF2LLC cells (Figure 6G). Our findings suggested that NETs promoted ferroptosis resistance in LLC cells via the YTHDF2‐SLC2A3 axis.

### 3.7 NETs attenuated CD8(+) T cells effects on ferroptosis of LLC cells

**FIGURE 7:**
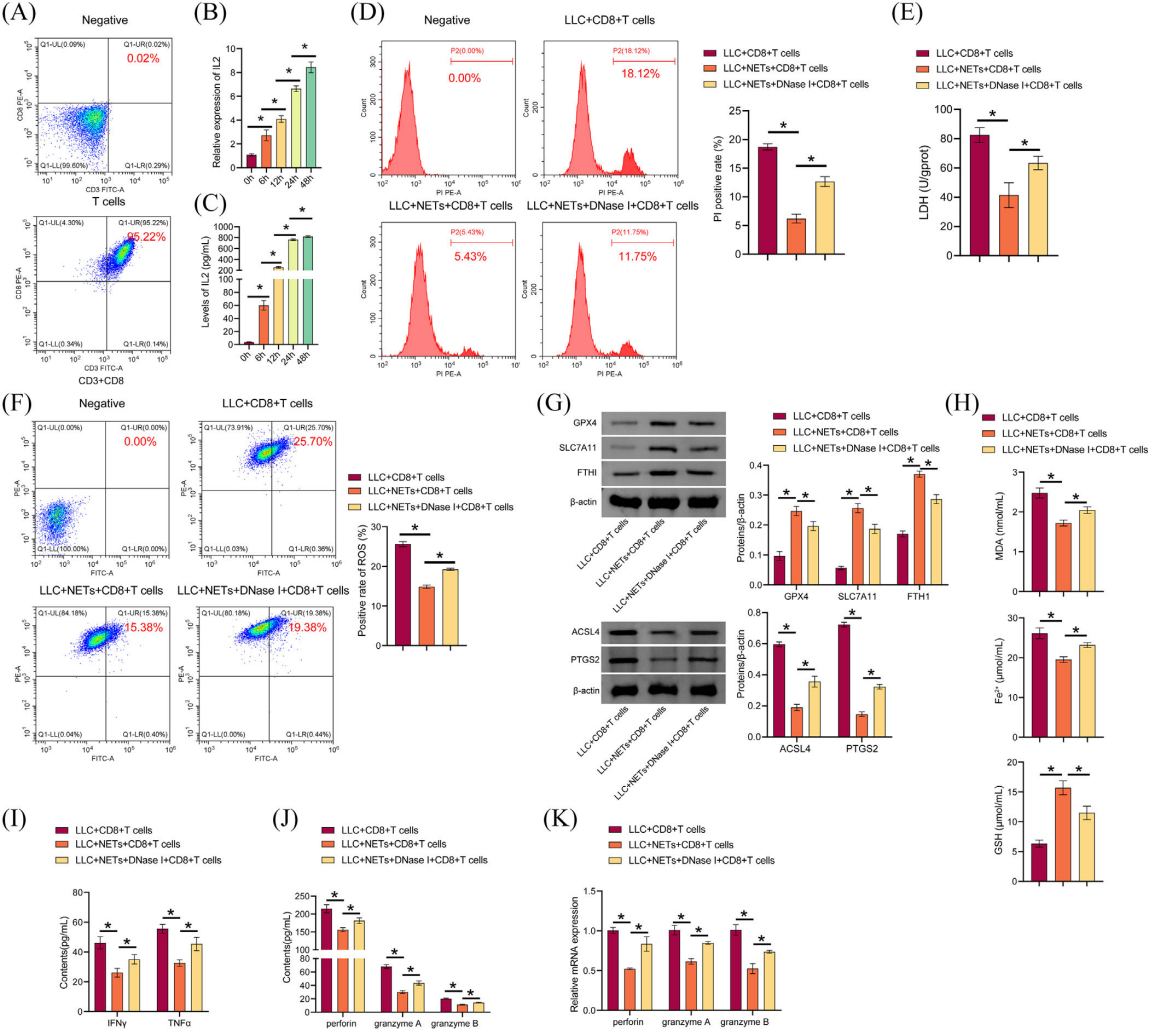
NETs attenuated CD8(+) T cells effects on ferroptosis of LLC cells. (A) Primary mouse T cell identification. (B) IL2 mRNA levels in T cells. (C) ELISA analysis of IL2 levels in T cell supernatant. (D) PI staining was used to detect LLC cell death by flow cytometry. (E) ELISA analysis of LDH levels. (F) The presence of lipid ROS was determined through the use of C11 BODIPY 581/591. (G) GPx4, SLC7A11, FTH1, ACSL4, and PTGS2 proteins expression. (H) Biochemical kits analysis of MDA, total Fe^2+^, and GSH levels. (I) ELISA analysis of IFNγ and TNF‐α levels in T cell supernatants. (J) ELISA detection of perforin, granzyme A, and granzyme B levels. (K) qRT‐PCR analysis of perforin, granzyme A, and granzyme B levels. *p < .05.

CD8(+) T cells coordinate ferroptosis and immunity responses in the TME.^38^ CD8(+) T cells are very important in cancer immunotherapy because they produce IFNγ, which activates the STAT1 signalling pathway, inhibits SLC7A11 expression and induces ferroptosis in tumour cells.^39^ Therefore, we further explored whether CD8(+) T cells mediated NETs effects on ferroptosis in LLC cells. First, anti‐CD3+anti‐CD28 costimulation was used to activate mouse primary CD3(+)CD8(+) T cells, and it was found that the proportion of CD3(+)CD8(+) positive cells was 95.22%, which was more than 90% (Figure 7A). In addition, IL2 secretion, which reflected T cell activation, increased with the extension of stimulation time (Figure 7B and C). Our results showed that primary mouse T cells were successfully activated. Activated CD8(+) T cells were cocultured with differentially treated LLC cells. The results showed that cell death and LDH levels were lower in the LLC+NETs+CD8+T cells group than in the LLC+CD8+T cells group. Compared with LLC+NETs+CD8+T cells, cell death and LDH levels in LLC+NETs+DNase I+CD8+T cells were increased (Figure 7D and E). Lipid ROS production was lower in the LLC+NETs+CD8+T cells group than in the LLC+CD8+T cells group. Compared to LLC+NETs+CD8+T cells, the production of lipid ROS in the LLC+NETs+DNase I+CD8+T cells was increased (Figure 7F). Compared to the LLC+CD8+T cells group, GPX4, SLC7A11, and FTH1 levels in the LLC+NETs+CD8+T cells group were upregulated, while ACSL4 and PTGS2 levels were downregulated. Compared with the LLC+NETs+CD8+T cells group, GPX4, SLC7A11, and FTH1 levels in the LLC+NETs+DNase I+CD8+T cells group were downregulated, while ACSL4 and PTGS2 levels were upregulated (Figure 7G). Compared with the LLC+CD8+T cells, MDA and total Fe^2+^ levels in the LLC+NETs+CD8+T cells were decreased, while GSH levels were increased. Compared with the LLC+NETs+CD8+T cells group, MDA and total Fe^2+^ levels in the LLC+NETs+DNase I+CD8+T cells group were increased, while GSH levels was decreased (Figure 7H). Compared with the LLC+CD8+T cells group, IFNγ and TNF‐α levels in the LLC+NETs+CD8+T cells group was downregulated. Compared with the LLC+NETs+CD8+T cells group, IFNγ and TNF‐α levels in the LLC+NETs+DNase I+CD8+T cells group were upregulated (Figure 7I). Compared with the LLC+CD8+T cells group, perforin, granzyme A, and granzyme B levels in the LLC+NETs+CD8+T cells group was inhibited. Compared with the LLC+NETs+CD8+T cells group, perforin, granzyme A, and granzyme B levels in the LLC+NETs+DNase I+CD8+T cells group was increased (Figure 7J and K). Our results showed that NETs attenuated CD8(+) T cells effects on ferroptosis in LLC cells.

### 3.8 Coinhibition of NETs and YTHDF2 promoted ferroptosis, increased CD8(+) T cell activity, and inhibited LUAD growth

**FIGURE 8:**
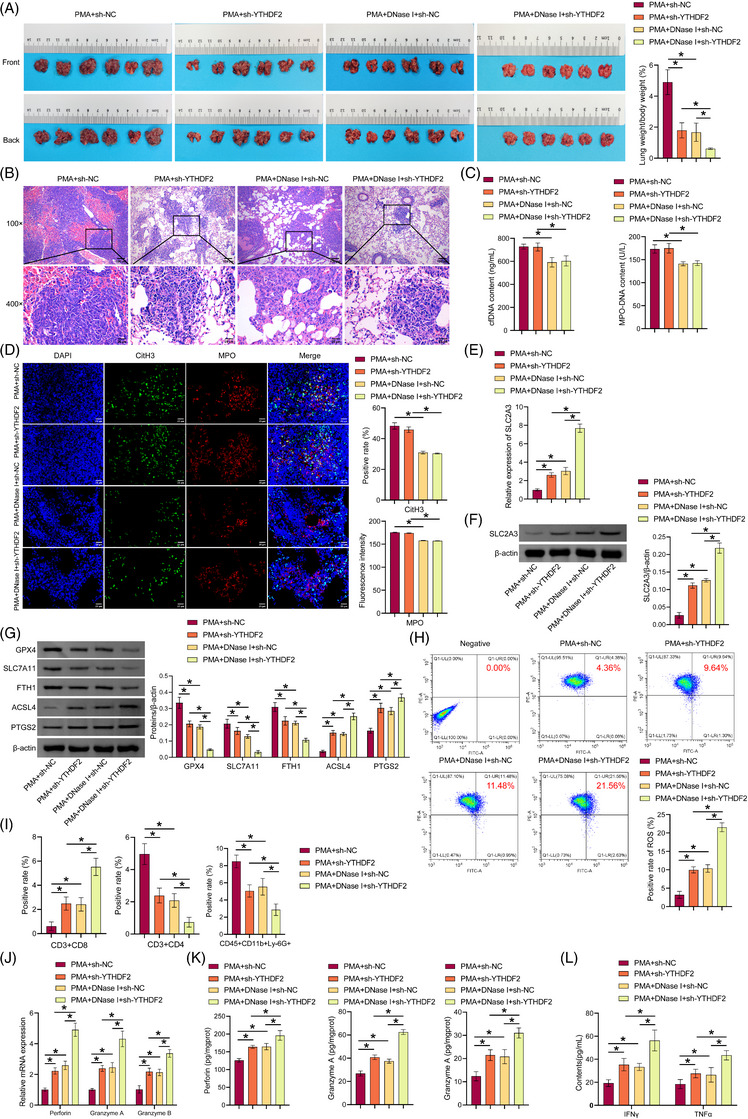
Coinhibition of NETs and YTHDF2 on ferroptosis, CD8(+) T cell activity, and LUAD growth. (A) Lung tumour images of mice and lung weight/body weight. (B) Tissue morphology was observed by H&E staining. (C) IF detection of CitH3 and MPO colocalisation in tumour. (D) Test kit for cfDNA and MPO‐DNA content in serum. (E) qRT‐PCR analysis of SLC2A3 levels in tumour. (F) Western blot analysis of SLC2A3 levels in tumour. (G) GPx4, SLC7A11, FTH1, ACSL4, and PTGS2 proteins expression. (H) The presence of lipid ROS was determined through the use of C11 BODIPY 581/591. (I) A flow cytometry analysis was conducted on CD3(+)CD8(+)T cells, CD3(+)CD4(+)T cells, CD45(+)CD11b(+)Ly‐6G(+)neutrophils. (J) qRT‐PCR and (K) ELISA detection of perforin, granzyme A, and granzyme B levels. (L) ELISA analysis of serum IFNγ and TNF‐α expression. *p < .05.

We then investigated in vivo coinhibition of NETs and YTHDF2 in mediating ferroptosis and CD8(+) T cells role on LUAD growth. Compared with the PMA+sh‐NC group, the positive rate of GFP in lung tissue was reduced in the PMA+sh‐YTHDF2 and PMA+DNase I+sh‐NC groups, and the positive rate of GFP in lung tissue in the PMA+DNase I+sh‐YTHDF2 group was further reduced (Figure S2C). Compared with the PMA+sh‐NC group, the lung weight‐to‐body weight ratio of mice decreased in the PMA+sh‐YTHDF2 and PMA+DNase I+sh‐NC groups, which was further reduced in the PMA+DNase I+sh‐YTHDF2 group (Figure 8A). H&E staining showed that sh‐YTHDF2 and DNase I reduced lung tumour nodules and tumour cells number, and increased necrotic cells number, with a superimposed effect (Figure 8B). DNase I decreased cfDNA and MPO‐DNA content in the serum and reduced the colocalisation of CitH3 and MPO in tumours. The results demonstrated that sh‐YTHDF2 had no notable impact on NET formation (Figure 8C and D). Sh‐YTHDF2 and DNase I promoted SLC2A3 expression in tumours and had a superimposed effect (Figure 8E and F). Sh‐YTHDF2 and DNase I inhibited GPX4, SLC7A11, and FTH1 expression in the tumour, while increasing ACSL4 and PTGS2 levels and had a superimposed effect (Figure 8G). Sh‐YTHDF2 and DNase I increased the production of lipid ROS in tumours and had a superimposed effect (Figure 8H). Sh‐YTHDF2 and DNase I increased CD3(+)CD8(+)T cells number and decreased CD3(+)CD4(+)T cells and CD45(+)CD11b(+)Ly‐6G(+)neutrophil number. Sh‐YTHDF2 and DNase I showed a superposition effect (Figure 8I and S2A). Sh‐YTHDF2 and DNase I increased perforin, granzyme A, and granzyme B expression. Sh‐YTHDF2 and DNase I showed a superposition effect (Figure 8J and K). Sh‐YTHDF2 and DNase I promoted IFNγ and TNF‐α expression in the serum and had a superimposed effect (Figure 8L). Our findings suggested that coinhibition of NETs and YTHDF2 promoted ferroptosis, increased CD8(+) T cell activity, and inhibited LUAD growth.

## 4 DISCUSSION

In this study, our results showed increased CitH3 and MPO expression in lung tissue and elevated cfDNA and MPO‐DNA levels in the serum of LUAD patients, indicating increased NETs formation in LUAD. In mice, PMA increased the formation of NETs and CD3(+)CD4(+)T cells number, decreased CD3(+)CD8(+)T cells number in tumour and perforin, granzyme A, granzyme B, IFNγ, and TNF‐α levels, and promoted LUAD growth and the number of lung tumour nodules, suggesting that PMA promoted NETs formation, inhibited CD8(+) T cells activity, and promoted LUAD growth. DNase I partially reversed the effect of PMA, reduced the formation of NETs, increased the activity of CD8(+) T cells, and inhibited LUAD growth. NETs promoted LLC cell proliferation and migration in vitro, whereas DNase I reversed the effects of NETs. Erastin inhibited the proliferation and migration of LLC cells and promoted ferroptosis. NETs partially reversed Erastin effects. Further results showed that NETs promoted LLC cell proliferation and migration, and inhibited ferroptosis by promoting YTHDF2‐mediated SLC2A3 mRNA degradation. Sh‐YTHDF2 partially reversed NETs effects on LLC cells, while si‐SLC2A3 partially reversed the effect of sh‐YTHDF2 on LLC cells. In addition, NETs inhibited LLC cell ferroptosis by inhibiting CD8(+) T cell activity. Sh‐YTHDF2 and DNase I inhibited NETs formation in tumour, increased CD8(+) T cells activity, and inhibited LUAD growth. In summary, our results suggested that NETs promoted YTHDF2‐mediated SLC2A3 mRNA degradation, and inhibited ferroptosis and CD8(+) T cell activity, thereby promoting LUAD.

Previous studies have shown that NETs impede the interaction between cytotoxic T lymphocytes, natural killer cells, and adjacent tumour cells, thereby reducing the efficacy of immunotherapy.^40^ The mechanism of neutrophil recruitment and NETs formation in the metastatic microenvironment is promoted by liver cancer through a reduction in HRG secretion, thus facilitating lung metastasis.^30^ Our results indicated an increase in NETs formation in LUAD, suggesting that NETs formation may contribute to LUAD progression. CD8(+) T cells are key immune effector cells in anti‐tumour immunity.^41^ Studies have shown that MTSS1 is downregulated in LUAD, leading to upregulation of PD‐L1, impaired function of CD8(+) T cells and accelerated tumour progression.^42^ Consumption of FBXO38 significantly increases the abundance of FGL1, inhibits CD8(+) T cell infiltration, and enhances tumour immune escape, thereby promoting NSCLC progression.^43^ Our results suggested that PMA increased NETs formation, decreased CD8(+) T cells activity, and promoted LUAD growth. DNase I partially reversed the effect of PMA, inhibited NETs formation, increased CD8(+) T cells activity, and inhibited LUAD growth. NETs promoted LLC cell proliferation and migration, while DNase I reversed these effects. In addition, NETs inhibited LLC cell ferroptosis by inhibiting CD8(+) T cell activity. Our results suggested that NETs promoted LUAD growth by decreasing CD8(+) T cell activity.

The significance of m6A RNA modification has been substantiated in a multitude of tumour types.^44^ Studies have shown that IGF2BP3 induces ferroptosis resistance and promotes LUAD.^45^ METTL3 promotes metastatic growth and inhibits ferroptosis in LUAD cells through the stabilisation of the SLC7A11 m6A modification.^17^ Downregulation of LINC00641 modified by YTHDC1 in m6A readers increased arachidonic acid metabolism and promoted ferroptosis.^46^ As an m6A‐modified reading protein, YTHDF2 mediates mRNA metabolism by regulating the translation and stability. Abnormal expression or function of YTHDF2 is strongly correlated with the onset and metastasis.^47^ KIAA1429 has been demonstrated to facilitate the development of LUAD through a mechanism dependent on m6A‐mediated regulation of the BTG2 gene, which involves interaction with the YTHDF2 protein.^48^ YTHDF2 facilitates the growth and metastasis of LUAD cells.^36^ Our results suggested that NETs enhanced the proliferation and migration abilities of LLC cells and inhibited ferroptosis by promoting YTHDF2‐mediated SLC2A3 mRNA degradation. Sh‐YTHDF2 partially reversed NETs effects on LLC cells, decreased the proliferation and migration abilities of LLC cells, and induced ferroptosis. Si‐SLC2A3 partially reversed the effect of sh‐YTHDF2 on LLC cells. In addition, sh‐YTHDF2 and DNase I inhibited NETs formation in tumour, increased CD8(+) T cells activity, and inhibited LUAD growth. Our results are consistent with previous studies. Inhibitors targeting YTHDF2 may offer a novel therapeutic approach to the treatment of LUAD.

However, there are some limitations. Lack of relevant experimental data to clarify the possible mechanism of NETs formation in LUAD. NETs are induced in cancer through various mediators. Ex vivo, inflammatory molecules released from cancer cells [e.g., IL‐8/CXCL8, granulocyte colony‐stimulating factor (G‐CSF), CXCL1, CXCL2, Cathepsin C, and Toll‐like receptor (TLR) ligands] can induce NETs.^49^ Tumour‐derived exosomes can induce NETs, thereby promoting tumour growth.^50^ Cancer‐associated fibroblast derived amyloid beta promotes tumour growth through inducing the formation of NETs.^51^ It is important to explore further the underlying molecular mechanisms that lead to NET formation in LUAD mediated by tumour‐derived exosomes and cancer‐associated fibroblasts. Due to its unophysiologic characteristics and potential risks, it is necessary to take into account the unnatural effects that PMA may have on experimental results. It is important to explore the mechanism in LUAD using more physiologically and clinically relevant NETs stimulation. Considering the limited funds, in this study, we did not apply additional YTHDF2 inhibitors (DC‐Y13 and some derivatives) to clarify further coinhibition of NETs and YTHDF2 promoted ferroptosis, increased CD8(+) T cell activity, and inhibited LUAD growth. In future projects, it will be important to further explore the potential molecular mechanisms of YTHDF2 in LUAD using YTHDF2 inhibitors (DC‐Y13 and some derivatives). In addition, NETs are usually present in tissues from patients with advanced tumours, and our tissue samples are in the middle and advanced stages (stage IIIA). We speculate that a more typical NETs network structure may be present in advanced patients after stage IIIA. However, because advanced patients are usually treated conservatively, tissue samples are difficult to collect, which is a limitation of our study.

## 5 CONCLUSION

Our findings indicated that NETs promoted LUAD growth by mediating the stability of m6A‐mediated SLC2A3 mRNA‐induced ferroptosis resistance and CD8(+) T cell inhibition.

## AUTHOR CONTRIBUTIONS

Bolin Chen: guarantor of integrity of the entire study, literature research, study design and manuscript review. Li Xu: study design, experimental studies and manuscript editing. Yi Kong: statistical analysis and validation. Kang Li and Jia Li: data analysis and software. Fang Xu, Yan Xu, Shuzhi Liang: data acquisition. All authors read and approved the final manuscript.

## CONFLICT OF INTEREST STATEMENT

The authors declare no conflicts of interest.

## FUNDING

This work was supported by Hunan Provincial Natural Science Foundation of China (No. 2024JJ5238), the High‐level Talents Project of Hunan Health Commission (No. [2023]32‐131), and the Wu Jieping Medical Foundation (320.6750.2023‐05‐11).

## ETHICS STATEMENT

The study was approved by the Medical Ethics Committee of Hunan Cancer Hospital (Number: SBQLL‐2022‐113). All the data and material have been performed following the Declaration of Helsinki and conformed to relevant aspects of the ARRIVE guidelines. All the information about the study will be fully explained to the subjects by the researchers. All patients signed written informed consent. Informed consent was obtained from the participants.

All experimental procedures and animal handling were performed with the approval of the Animal Care and Use Committee of the Hunan Cancer Hospital (Number: 2022–084), in accordance with the National Institutes of Health Guide for the Care and Use of Laboratory Animals.

## Supporting information



Supporting Information

Supporting Information

Supporting Information

## Data Availability

The datasets used and analysed during the current study are available from the corresponding author on reasonable request.
