# Supplementary material for: Neutrophil extracellular traps promote growth of lung adenocarcinoma by mediating the stability of m6A‐mediated SLC2A3 mRNA‐induced ferroptosis resistance and CD8(+) T cell inhibition
Source: Clin Transl Med. 2025 Jan 26;15(2):e70192. doi: 10.1002/ctm2.70192 (PMC11769710; doi:10.1002/ctm2.70192)
Supplement: Supplementary file 1 — Supporting Information [file CTM2-15-e70192-s003.docx]

**Supplementary information**

**Supplementary Methods.** The experimental protocols used in this study.

**
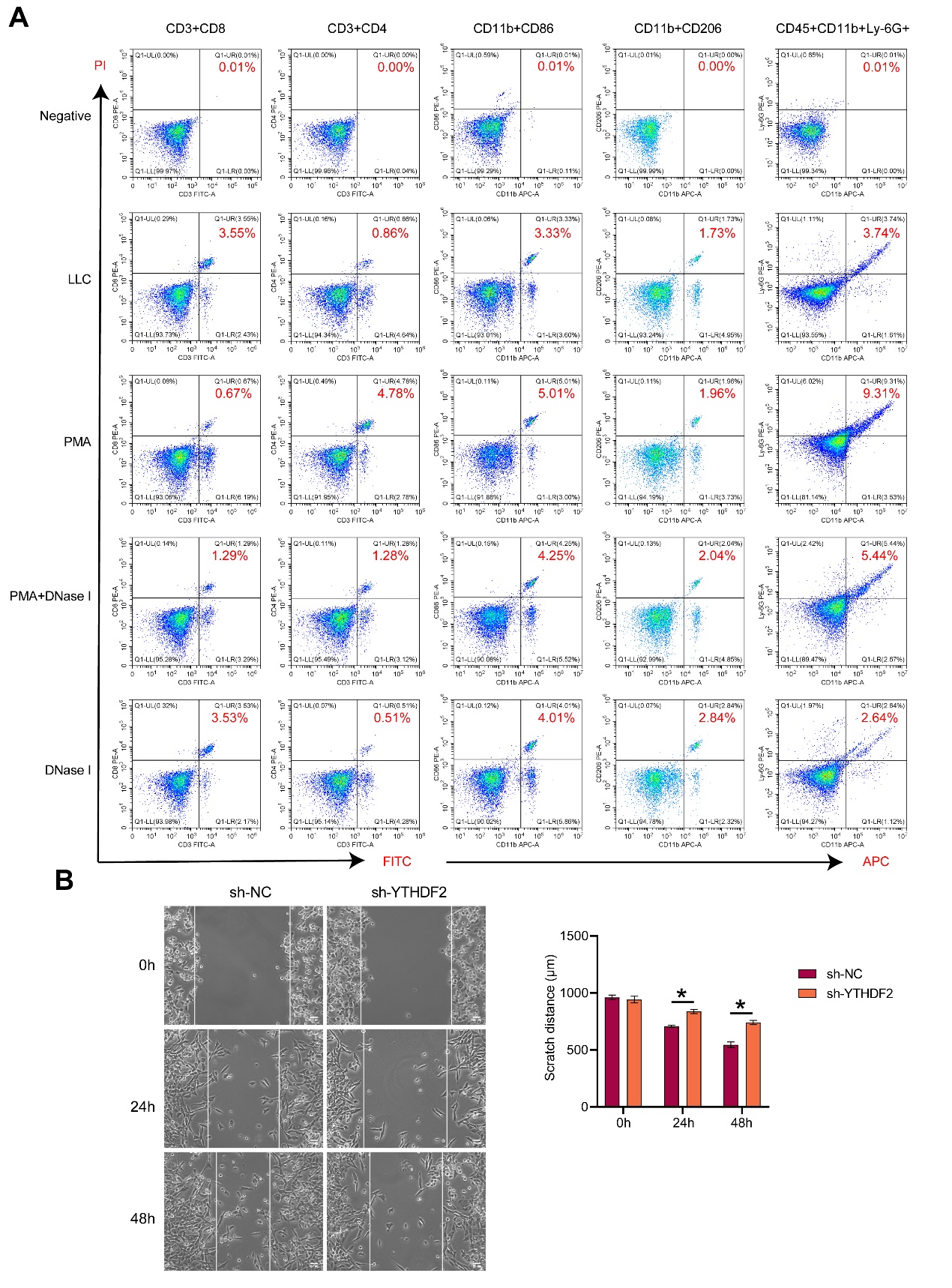
**

**Figure S1.** (A) A flow cytometry analysis was conducted on T cells, neutrophils, M1 macrophages, and M2 macrophages. (B) Scratch detection of cell migration capacity. * p < 0.05.

**
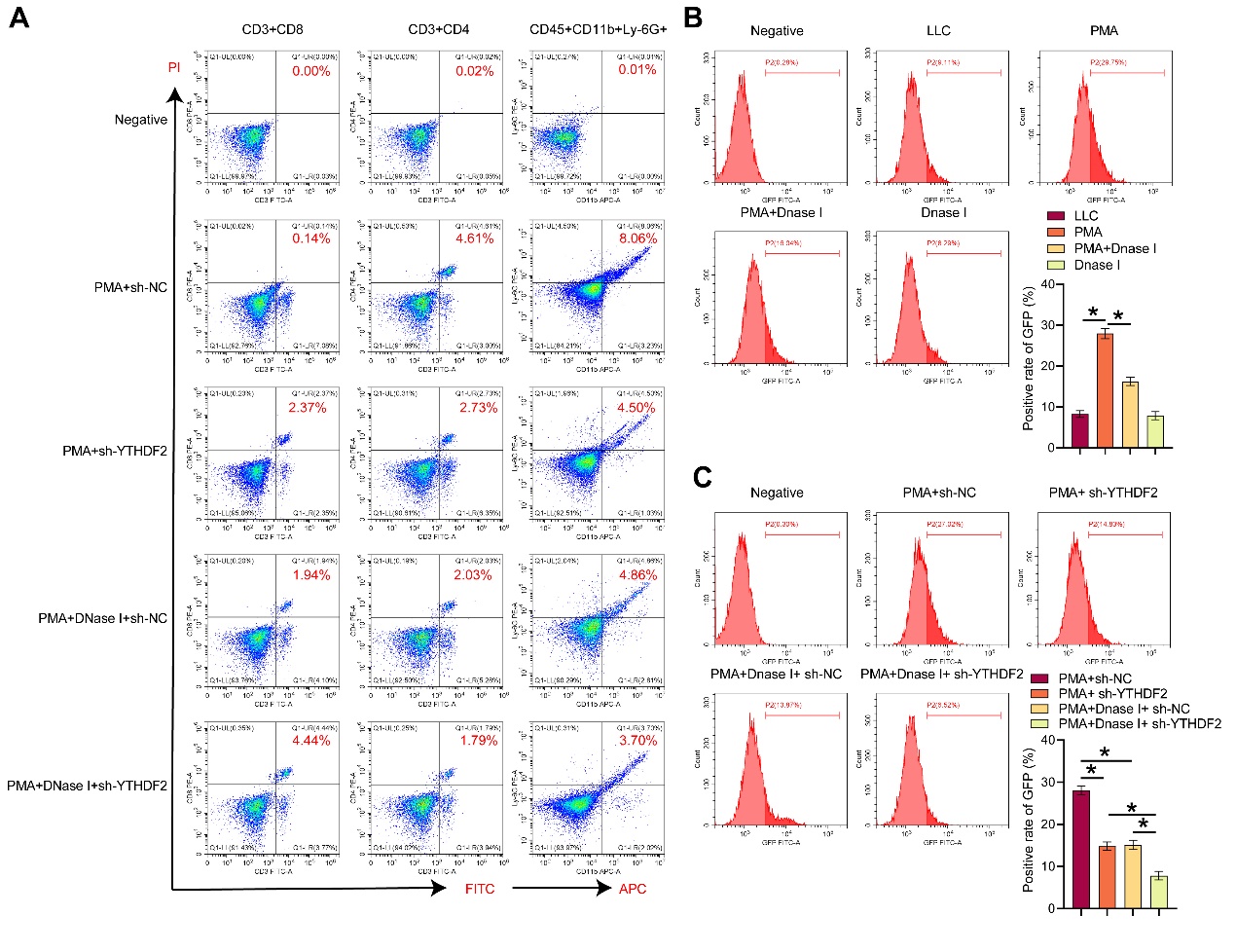
**

**Figure S2.** (A) A flow cytometry analysis was conducted on T cells and neutrophils. (B and C) Positive rates of GFP in lung tissue were analyzed by flow cytometry. * p < 0.05.

**
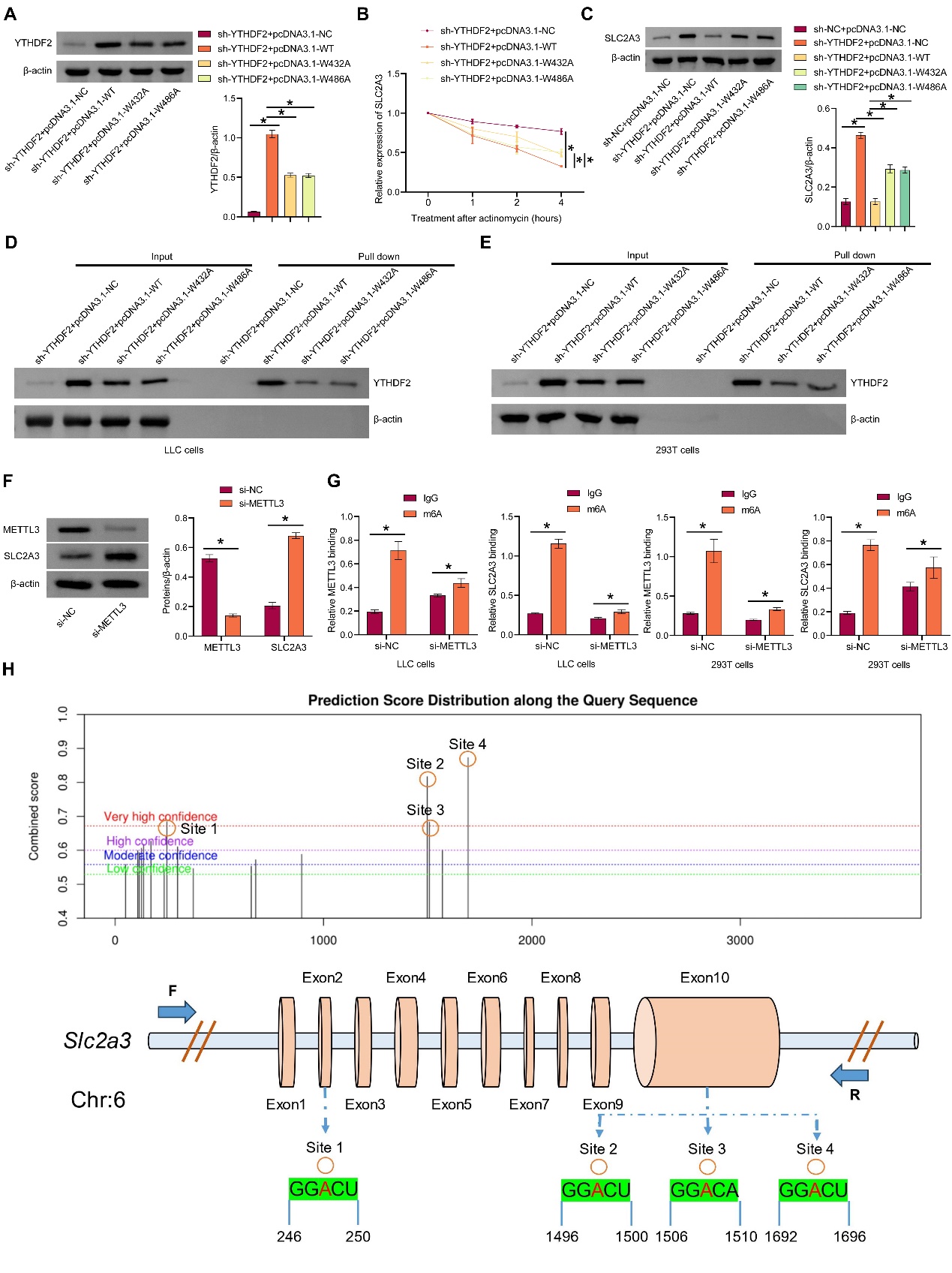
**

**Figure S3.** An empty vector (pcDNA3.1) or vectors encoding wild-type (WT) or mutant YTHDF2 (W432A and W486A) were transferred into cells that had been transduced with sh-NC/sh-YTHDF2 for 24 h. (A) YTHDF2 expression was confirmed using a western blot. (B) SLC2A3 mRNA stability was ascertain by qRT-PCR after treating with 5 μg/mL actinomycin D. (C) YTHDF2 expression were analyzed by western blot. (D and E) RNA pull-down was used to assess the combination of SLC2A3 mRNA and YTHDF2. (F) Western blot was employed to ascertain SLC2A3 and METTL3 levels. (G) MeRIP-qPCR analysis of SLC2A3 mRNA in si-NC or si-METTL3-cells. (H) Bioinformatic prediction of m6A modification sites in SLC2A3 mRNA using the SRAMP database. * p < 0.05.
