# Supplementary material for: Neutrophil extracellular traps promote growth of lung adenocarcinoma by mediating the stability of m6A‐mediated SLC2A3 mRNA‐induced ferroptosis resistance and CD8(+) T cell inhibition
Source: Clin Transl Med. 2025 Jan 26;15(2):e70192. doi: 10.1002/ctm2.70192 (PMC11769710; doi:10.1002/ctm2.70192)
Supplement: Supplementary file 2 — Supporting Information [file CTM2-15-e70192-s001.docx]

**Materials and methods**

**Neutrophil extraction**

A peripheral blood neutrophil extraction kit was employed for the isolation of neutrophils from the peripheral blood of the healthy donors. Isolated primary neutrophils were maintained in RPMI 1640 medium with the additional supplementation of 10% FBS. Giemsa staining and Trypan blue activity assay were used to determine neutrophil purity (> 98%) and activity (> 95%).

Neutrophils were isolated from mouse bone marrow using a mouse Neutrophil Isolation Kit (Miltenyi, #130-097-658). Briefly, a 50 µL neutrophil biotin-antibody mixture was added to a 200 µL cell suspension (the total number of cells in the magnetically activated cell sorting (MACS) buffer was 5×10^7^). Following this addition, the solution was incubated at 4℃ for 15 min. After washing twice with MACS buffer, 100 µL of magnetic microbeads were added to 400 µL of the cell suspension. Subsequently, magnetic separation was conducted using an LS column and MidiMACS separator (Miltenyi).

**CD8+T cell purification**

CD8(+) T cells were isolated from the spleens of C57BL/6J mice using a MojoSort™ mouse CD8(+) T cell isolation kit (480008, BioLegend), following the manufacturer’s instructions.

**Lipid ROS analysis**

The cells were inoculated into 12-well plates and incubated with 1 mL of fresh medium containing 5 μM BODIPY 581/591 C11 (Invitrogen) for 30 min. The cells were then digested with trypsin, cleaned, and re-suspended in PBS (0.5 mL). Lipid ROS production was analyzed using flow cytometry (BD Biosciences).

**Hematoxylin and eosin (H&E) staining**

The tissues were fixed in 4% paraformaldehyde for 24 h. The samples were dehydrated by gradient ethanol, and xylene was transparent. The specimens were then embedded in paraffin wax. The sections 4μm thick were prepared using a paraffin microtome (YD-315, Yidi, China). The sections were baked in an oven at 60℃ for 8 h. The tissues were dewaxed and rehydrated by xylene and gradient ethanol. The tissues were stained with hematoxylin and eosin, respectively. The specimens were dehydrated by gradient ethanol. Xylene was transparent. The results were observed under an optical microscope (BA210T, MOTIC, Singapore) after the neutral gum was sealed.

**Flow cytometry**

To analyze immune cell infiltration in tumors, tumor tissues were ground into single cells, filtered with a 70 μm cell filter, and purified by Percoll gradient (Yeasen, Shanghai). The cells (2 × 10^6^) were incubated with appropriate antibodies at room temperature for 30 min. After washing twice in PBS, the samples were analyzed using flow cytometry (BD Biosciences, San Jose, CA). The antibodies used included rat FITC-CD3 (11-0032-82, eBioscience, USA), rat PE-CD8a (12-0081-82, eBioscience, USA), rat PE-CD4 (12-0041-82, eBioscience, USA), rat FITC-CD45 (11-0451-82, eBioscience, USA), rat APC-CD11b (17-0112-82, eBioscience, USA), rat PE-CD86 (12-0862-82, eBioscience, USA), rat PE-CD206 (12-2061-82, USA) eBioscience, USA), rat PE-Ly-6G (12-9668-82, eBioscience, USA), FITC-rat IgG2b kappa Isotype Control (11-4031-82, eBioscience, USA), PE-rat IgG2b kappa Isotype Control (12-4031-82, eBioscience, USA), PE-rat IgG2a kappa Isotype Control (12-4321-80, eBioscience, USA), and APC-rat IgG2b kappa Isotype Control (17-4031-82, eBioscience, USA).

**The assessment of cell death by Propidium iodide (PI) staining**

The PI Kit (40710ES03, Yeasen, China) was used to evaluate cell death. The cells were harvested, resuspended in 100 μL of binding buffer containing 5 μL of PI (50 μg/mL), and incubated in the dark at 4 ℃ for 15 min. Flow cytometry (BD Biosciences, San Jose, CA) was used to assess cell death.

**Immunofluorescence (IF)**

Such as H&E staining to obtain tissue sections. The sections were placed in the periodate solution to inactivate endogenous enzymes. 5% BSA was closed at 37℃ for 1 h and incubated with a primary antibody, including CitH3 (ab281584, 1:50, abcam, UK) and MPO (66177-1-Ig, 1:50, Proteintech, USA) at 4℃ overnight. The samples were incubated with diluted Goat anti-rabbit IgG (H+L) Secondary Antibody, Alexa Fluor 488 (AWS0005a, 1:100, Abiowell, China) and Goat anti-mouse IgG (H+L) Secondary Antibody, Alexa Fluor 594 (AWS0004a, 1:100, Abiowell, China) at 37℃ for 90 min, washed with PBS for 5 min, 3 times. DAPI working solution was used at 37℃, nucleated for 10 min, and washed with PBS for 5 min, 3 times. The buffered glycerin tablets were sealed and observed under a fluorescence microscope (BA410T, MOTIC, Singapore).

**Quantitative real‐time quantitative polymerase chain reaction (qRT-PCR) analysis**

Total RNA from tissues and cells was extracted using Trizol reagent (Invitrogen, Carlsbad, CA, USA). The GoScript Reverse Transcription System (Promega, Madison, Wis) was used to generate combinational DNA. The cDNA template was amplified by real-time RT-PCR using the SYBR Premix Dimmer Eraser kit (TaKaRa). Gene expression was normalized to β-Actin expression. The relative expression fold change of mRNAs was calculated by the 2^-ΔΔCt^ method. Primers used in this study are listed in Supplementary Table 2.

**Western blot**

The total cellular proteins were prepared using RIPA cell lysis buffer (Cell Signaling Technology) supplemented with protease inhibitors. The lysates were then collected and subjected to ultrasonication and centrifugation. The supernatants were collected, and protein content was determined by BCA assay. Equal amounts (30-50 μg) of proteins were applied to an 8-12% SDS-polyacrylamide separating gel and transferred to a PVDF Immobilon-P membrane (Millipore). The membrane was blocked with 5% skim milk in TBST and then probed with indicated primary antibodies with gentle shaking at 4℃ overnight. The membrane was incubated with secondary antibody HRP Goat-anti-mouse IgG(SA00001-1, 1:5000, proteintech, USA) or HRP Goat-anti-rabbit IgG(SA00001-2, 1:6000, proteintech, USA) at room temperature for 90 min. Antibody-bound proteins were detected by ECL (enhanced chemiluminescence) Western Blotting Substrate (Pierce, Rockford, IL). The band intensity of western blotting and the normalization were analyzed using the Image J program (National Institutes of Health, Bethesda, MD). The primary antibody information used in the study is shown in Supplementary Table 3.

**Enzyme-linked immunosorbent assay (ELISA)**

ELISA kit was used to measure free DNA (cfDNA), MPO-DNA, interferon-gamma (IFNγ), tumor necrosis factor-alpha (TNF-α), perforin, granzyme A, granzyme B, IL-2, and LDH levels in serum and cell supernatants. The absorbance at 450 nm was then measured using a micro readout. cfDNA (DP710, TIANGEN, China), MPO-DNA (ml550432, ml110452, mlbio, China), IFNγ (CSB-E04578m, CUSABIO, China), TNF-α (CSB-E04741m, CUSABIO, China), perforin (CSB-E13429m, CUSABIO, China), granzyme A (CSB-E08717m, CUSABIO, China), granzyme B (CSB-E08720m, CUSABIO, China), IL-2 (CSB-E04627m, CUSABIO, China), and LDH (A020-2-2, Nanjing jiancheng Bioengineering Institute, China) ELISA kits were operated according to manufacturer's instructions.

**Detection of malondialdehyde (MDA), glutathione (GSH), and Fe^2+^**

The concentration of MDA and GSH in cell lysates/tissues were measured using the MDA assay kit (A003-1, Nanjingjiancheng, China) and microreduced GSH assay kit (A006-2, Nanjingjiancheng, China) according to the manufacturer's instructions. Fe^2+^ concentration was measured using an iron ion assay kit manufactured by the manufacturer (JL-T1255, Jianglaibio, China). The determination procedure was based on the kit protocol.

**Wound healing assay**

Wound healing assay was applied for measuring cell migration. A total of 1 × 10^5^ cells were inoculated in a 6-well plate, and when the cell confluence was about 90%, an artificial wound was created on the cell layer with a 200 µL gun tip. The wound healing was observed 24 h later, and imaging was performed under the microscope. The distance the cells travel was expressed as the percentage of cells that cover the initial wound boundary. The distance traveled by the cells was expressed as the percentage of cell coverage across the initial wound demarcation.

**5-Ethynyl-2'- deoxyuridine (EdU) assay**

The treated cells (2 × 10^4^ cells/well) were inoculated into a 24-well plate and the newly synthesized DNA was detected using the EdU assay kit (C10310-1, Ribobio, China) according to the manufacturer's instructions.

**Supplementary Table 1. The clinical characteristics of patients.**

| **Items** | **Lung adenocarcinoma** |
| --- | --- |
| Total | 10 |
| participants |  |
| Female (n, %) | 7 (70%) |
| Male (n, %) | 3 (30%) |
| Age (years, X±SD) | 65.5 ± 5.64 |
| Precise site (n, %) |  |
| Right lower lobe lung | 3 (30%) |
| Right middle lung | 1 (10%) |
| Right upper lobe lung | 4 (40%) |
| Left upper lobe lung | 2 (20.0%) |
| Size (cm, n, %) |  |
| ≥2*2*1.5 | 5 (50.0%) |
| ＜2*2*1.5 | 4 (40.0%) |
| TNM stage (n, %)  IIIA | 10 (100.0%) |

**Supplementary Table 2. Primers used in the study.**

| **Name** | **Sequence** |
| --- | --- |
| M-perforin | F TCTTGGTGGGACTTCAGCTT  R TGCTTGCATTCTGACCGAGT |
| M-granzyme A | F CTCCGTGGTGGAAAGGACTC  R AGAGGTGATGCCTCGCAAAA |
| M-granzyme B | F GAAGCCAGGAGATGTGTGCT  R GCACGTTTGGTCTTTGGGTC |
| M-DUSP1 | F CAGATTAGGAGCAGCGAGCA  R AAAGCGAAGAAGGAGCGACA |
| M-SLC2A3 | F CAGCTCCAGCAAGCAATTCG  R ACGTGCTAAGAAGGTGTCCG |
| M-SLC7A11 | F CATACTCCAGAACACGGGCAG  R AACAAAAGCCAGCAAAGGACCA |
| M-DDIT4 | F AAACAAAGGCTTAGGAGTCACAC  R ACAAACTGTCGGCCACCTC |
| M-IL2 | F GCCCCAAGGGCTCAAAAATG  R ACATAAACAGCAGGTCCAGT |
| M-β-actin | F ACATCCGTAAAGACCTCTATGCC  R TACTCCTGCTTGCTGATCCAC |

**Supplementary Table 3. Primary antibody used in the study.**

| **Name** | **Article number** | **Source** | **Dilution rate** | **Molecular weight** | **Company** | **Country** |
| --- | --- | --- | --- | --- | --- | --- |
| CitH3 | ab219407 | Rabbit | 1: 1000 | 15KDa | Abcam | UK |
| MPO | ab65871 | Rabbit | 1: 2000 | 84KDa | Abcam | UK |
| GPX4 | 67763-1-Ig | Mouse | 1: 1000 | 20-23KDa | Proteintech | USA |
| SLC7A11 | AWA00502 | Mouse | 1: 1000 | 55KDa | Abiowell | China |
| FTH1 | ab75972 | Rabbit | 1: 1000 | 21KDa | Abcam | UK |
| ACSL4 | 22401-1-AP | Rabbit | 1: 6000 | 79KDa | Proteintech | USA |
| PTGS2 | ab179800 | Rabbit | 1: 3000 | 69KDa | Abcam | UK |
| YTHDF1 | 17479-1-AP | Rabbit | 1: 4000 | 60KDa | Proteintech | USA |
| YTHDC1 | 29441-1-AP | Rabbit | 1: 10000 | 100KDa | Proteintech | USA |
| YTHDC2 | 27779-1-AP | Rabbit | 1: 4000 | 160KDa | Proteintech | USA |
| YTHDF2 | 24744-1-AP | Rabbit | 1: 4000 | 62KDa | Proteintech | USA |
| YTHDF3 | ab220161 | Rabbit | 1: 1000 | 73KDa | Abcam | UK |
| METTL3 | 15073-1-AP | Rabbit | 1: 1000 | 65-70KDa | Proteintech | USA |
| METTL14 | 26158-1-AP | Rabbit | 1: 5000 | 55-60KDa | Proteintech | USA |
| METTL16 | ab252420 | Rabbit | 1: 1000 | 64KDa | Abcam | UK |
| FTO | AWA55017 | Rabbit | 1: 1000 | 58KDa | Abiowell | China |
| DUSP1 | ab61201 | Rabbit | 1: 1000 | 39KDa | Abcam | UK |
| SLC2A3 | 20403-1-AP | Rabbit | 1: 5000 | 48-60KDa | Proteintech | USA |
| DDIT4 | ab191871 | Rabbit | 1: 1000 | 35KDa | Abcam | UK |
| β-actin | 66009-1-Ig | Mouse | 1: 5000 | 42KDa | Proteintech | USA |
