# Supplementary material for: Neutrophil extracellular traps promote growth of lung adenocarcinoma by mediating the stability of m6A‐mediated SLC2A3 mRNA‐induced ferroptosis resistance and CD8(+) T cell inhibition
Source: Clin Transl Med. 2025 Jan 26;15(2):e70192. doi: 10.1002/ctm2.70192 (PMC11769710; doi:10.1002/ctm2.70192)
Supplement: Supplementary file 3 — Supporting Information [file CTM2-15-e70192-s002.docx]

**Supplementary Table 1. The clinical characteristics of patients.**

| **Items** | **Lung adenocarcinoma** |
| --- | --- |
| Total | 10 |
| participants |  |
| Female (n, %) | 7 (70%) |
| Male (n, %) | 3 (30%) |
| Age (years, X±SD) | 65.5 ± 5.64 |
| Precise site (n, %) |  |
| Right lower lobe lung | 3 (30%) |
| Right middle lung | 1 (10%) |
| Right upper lobe lung | 4 (40%) |
| Left upper lobe lung | 2 (20.0%) |
| Size (cm, n, %) |  |
| ≥2*2*1.5 | 5 (50.0%) |
| ＜2*2*1.5 | 4 (40.0%) |
| TNM stage (n, %)  IIIA | 10 (100.0%) |

**Supplementary Table 2. Primers used in the study.**

| **Name** | **Sequence** |
| --- | --- |
| M-perforin | F TCTTGGTGGGACTTCAGCTT  R TGCTTGCATTCTGACCGAGT |
| M-granzyme A | F CTCCGTGGTGGAAAGGACTC  R AGAGGTGATGCCTCGCAAAA |
| M-granzyme B | F GAAGCCAGGAGATGTGTGCT  R GCACGTTTGGTCTTTGGGTC |
| M-DUSP1 | F CAGATTAGGAGCAGCGAGCA  R AAAGCGAAGAAGGAGCGACA |
| M-SLC2A3 | F CAGCTCCAGCAAGCAATTCG  R ACGTGCTAAGAAGGTGTCCG |
| M-SLC7A11 | F CATACTCCAGAACACGGGCAG  R AACAAAAGCCAGCAAAGGACCA |
| M-DDIT4 | F AAACAAAGGCTTAGGAGTCACAC  R ACAAACTGTCGGCCACCTC |
| M-IL2 | F GCCCCAAGGGCTCAAAAATG  R ACATAAACAGCAGGTCCAGT |
| M-β-actin | F ACATCCGTAAAGACCTCTATGCC  R TACTCCTGCTTGCTGATCCAC |

**Supplementary Table 3. Primary antibody used in the study.**

| **Name** | **Article number** | **Source** | **Dilution rate** | **Molecular weight** | **Company** | **Country** |
| --- | --- | --- | --- | --- | --- | --- |
| CitH3 | ab219407 | Rabbit | 1: 1000 | 15KDa | Abcam | UK |
| MPO | ab65871 | Rabbit | 1: 2000 | 84KDa | Abcam | UK |
| GPX4 | 67763-1-Ig | Mouse | 1: 1000 | 20-23KDa | Proteintech | USA |
| SLC7A11 | AWA00502 | Mouse | 1: 1000 | 55KDa | Abiowell | China |
| FTH1 | ab75972 | Rabbit | 1: 1000 | 21KDa | Abcam | UK |
| ACSL4 | 22401-1-AP | Rabbit | 1: 6000 | 79KDa | Proteintech | USA |
| PTGS2 | ab179800 | Rabbit | 1: 3000 | 69KDa | Abcam | UK |
| YTHDF1 | 17479-1-AP | Rabbit | 1: 4000 | 60KDa | Proteintech | USA |
| YTHDC1 | 29441-1-AP | Rabbit | 1: 10000 | 100KDa | Proteintech | USA |
| YTHDC2 | 27779-1-AP | Rabbit | 1: 4000 | 160KDa | Proteintech | USA |
| YTHDF2 | 24744-1-AP | Rabbit | 1: 4000 | 62KDa | Proteintech | USA |
| YTHDF3 | ab220161 | Rabbit | 1: 1000 | 73KDa | Abcam | UK |
| METTL3 | 15073-1-AP | Rabbit | 1: 1000 | 65-70KDa | Proteintech | USA |
| METTL14 | 26158-1-AP | Rabbit | 1: 5000 | 55-60KDa | Proteintech | USA |
| METTL16 | ab252420 | Rabbit | 1: 1000 | 64KDa | Abcam | UK |
| FTO | AWA55017 | Rabbit | 1: 1000 | 58KDa | Abiowell | China |
| DUSP1 | ab61201 | Rabbit | 1: 1000 | 39KDa | Abcam | UK |
| SLC2A3 | 20403-1-AP | Rabbit | 1: 5000 | 48-60KDa | Proteintech | USA |
| DDIT4 | ab191871 | Rabbit | 1: 1000 | 35KDa | Abcam | UK |
| β-actin | 66009-1-Ig | Mouse | 1: 5000 | 42KDa | Proteintech | USA |
